# Traditional and modern eating in older adults: a comparison between an urban and rural sample from Gujarat, Western India

**DOI:** 10.1080/21642850.2022.2116327

**Published:** 2022-08-29

**Authors:** Gudrun Sproesser, Rachana Bhangaokar, Matthew B. Ruby, Claude Fischler, Paul Rozin, Harald T. Schupp, Britta Renner

**Affiliations:** a Department of Psychology, University of Konstanz, Konstanz, Germany; b Department of Human Development & Family Studies, Maharaja Sayajirao University of Baroda, Vadodara, India; c Department of Psychology, Counselling and Therapy, La Trobe University, Albury-Wodonga, Australia; d Laboratoire d’Anthropologie Politique, Ecole des Hautes Etudes en Sciences Sociales, Paris, France; e Department of Psychology, University of Pennsylvania, Pennsylvania, PA, USA

**Keywords:** Nutrition transition, traditional and modern eating, older adults, Western India, rural

## Abstract

**Background:**

The present study aimed to investigate how often and to what degree older adults living in an area of Gujarat, Western India, enact traditional and modern eating behaviors. Specifically, we aimed to determine which facets of traditional eating are enacted rarely and which facets of modern eating are enacted often. Moreover, we hypothesized that urban older adults show a higher level of modern eating behaviors than rural older adults. Furthermore, we examined which traditional eating behaviors are more prevalent in rural older adults, and which are more prevalent in urban older adults.

**Methods:**

A trained research assistant administered a questionnaire in a face-to-face situation with 120 older adults in a rural and an urban area of Gujarat, Western India. Participants were asked how often and to what degree they perform 57 traditional and modern eating behaviors.

**Results:**

Overall, our sample of older Gujaratis reported a high level of traditional eating behaviors and a low level of modern eating behaviors. However, we also found, for example, a low level of the traditional eating facet of men getting preferential treatment and a high level of the modern eating facet of food being readily available. Moreover, most modern eating facets were more pronounced in the urban than in the rural sample. This was also the case for half of all traditional eating facets.

**Conclusion:**

Our sample of older adults living in an area of Gujarat displayed more modern eating behaviors in urban than in rural areas. At the same time, however, the urban sample showed also more traditional eating behaviors, such as eating more fruits, possibly because of better food availability. Altogether, results might hint at some signs of modernization among older adults in this area of Gujarat with regard to changing gender roles and better food availability.

## Introduction

Traditional eating behavior constitutes an important element of cultural heritage and identity (Guerrero et al., 2010; Tartakovsky & Abu Kheit, 2017), with cultural identity being associated with well-being (Usborne & Taylor, 2010). However, across the world, there has been a transition towards modern eating (e.g. Popkin & Ng, 2022), which might carry the risk of identity confusion (Arnett, 2002; Jensen & Arnett, 2012). Especially in middle-income countries, modern eating is rapidly increasing (e.g. Monteiro et al., 2013). India, one of the most populated nations in the world, is one example of such a middle-income country that is marked by rapidly shifting diets (e.g. Law et al., 2020).

With regard to traditional and modern eating behavior in India, most literature to date focuses only on nutrients or food groups, such as carbohydrates or cereals (e.g. Law et al., 2020; Misra et al., 2011; Siddiqui et al., 2019). In contrast, approaches from psychology have argued that behavior is often multidimensional (e.g. Renner et al., 2015). In line with this, a recent overview paper suggested that traditional and modern eating behavior goes beyond the consumption of certain nutrients or food groups. Specifically, Sproesser et al. (2019) compiled 106 behaviors that were regarded to be part of traditional and modern eating behavior in previous literature or in expert discussions, combining international and interdisciplinary perspectives. Importantly, what constitutes traditional and modern eating was considered to be subject to human evaluation, as it is often impossible to determine from actual measures how much change or what absolute level then and now is necessary to classify a behavior as traditional or modern. These behaviors were systematized, rendering three hierarchical levels of specificity. In line with wordings from personality assessment (e.g. Costa & McCrae, 1995), the 106 behaviors in the lowest level were called *facets*, denoting specific aspects within twelve *subdimensions* (the medium level). Among these subdimensions were, for example, *where*, *when*, and *with whom* people eat. The highest level was made up of the two major *dimensions* what and how people eat.

As evaluations of what constitutes traditional and modern eating behavior vary across cultures, Sproesser et al. (2022) asked a sample of 550 Indians from four regions (North, East, South, West) to rate the traditionality and modernity of a condensed set of 86 facets. Results from the four regions showed a highly similar pattern and were, thus, aggregated, revealing 63 facets that were perceived to be part of traditional and modern eating behavior in India. These facets cover all twelve subdimensions. In sum, for a comprehensive understanding of traditional and modern eating, more facets of eating behavior need to be taken into account than just nutrients and food groups.

The extent of traditional and modern eating differs across subpopulations in a country (Popkin et al., 2012; Sengupta et al., 2015). Older adults are a particularly interesting subpopulation in India, as the share of the population above 60 years is rapidly growing (Shetty, 2002). Despite this, older adults are rarely targeted in the literature (Schrock et al., 2017). Hence, we know little about which facets of eating behavior are already affected by modernization trends in older Indians. Still, one might speculate that, overall, older Indians show a high level of traditional eating and a low level of modern eating. Specifically, with regard to nutrients and food groups, older people are thought to show a relatively high level of traditional and a low level of modern eating (Pingali & Khwaja, 2004; Popkin et al., 2012). Also, older people have been shown to display a comparatively high level of food neophobia (Siegrist et al., 2013) and a low consumption of modern foods, such as sugar-sweetened beverages (Imamura et al., 2015).

Moreover, one might expect rural older Indians to show fewer signs of modern eating than urban older Indians. Specifically, regarding nutrients and food groups, researchers have concluded that modern eating is more pronounced in urban than in rural areas, but they have not identified a particular age group (Hawkes et al., 2017; Popkin et al., 2012; Shetty, 2002). In line with this difference, modern eating facets, such as eating outside the home and eating processed and fast foods, were higher in urban Indians than in rural Indians (d’Amour et al., 2020; Pandey et al., 2020; Smith, 2015). Differing socio-economic and demographic factors, such as higher income and smaller urban household size have been proposed as explanations for these differences (d’Amour et al., 2020). Also, urbanization has separated a large part of the world’s population from the direct production of foods (Hawkes et al., 2017).

When it comes to traditional eating facets, a less clear picture emerges. If one assumes that more modern eating goes along with less traditional eating, urban people should engage in fewer traditional eating behaviors than rural people. However, studies have revealed conflicting results. For instance, urban Indians have been reported to consume more dairy than rural Indians (Pandey et al., 2020; Shetty, 2002; Siddiqui et al., 2019), with dairy consumption having been rated as traditional eating behavior in India (Sproesser et al., 2022). Hence, the question arises which facets of traditional eating behavior are more pronounced in rural older adults, and which facets are more pronounced in urban older adults.

### The present study

The present study comprises a first step towards the investigation of how often and to what degree older Indians show traditional and modern eating behaviors. As there might be regional differences, we took an area of Gujarat, Western India, as a starting point. We aimed for a broad assessment of traditional and modern eating, including multiple facets as well as both what and how people eat (cf., Sproesser et al., 2019, 2022). There were three main questions with regard to older adults living around the city of Vadodara in Gujarat:

1. Which traditional eating behaviors are performed rarely and which modern eating behaviors are performed often?
2. Are modern eating behaviors more pronounced among urban than rural older adults?
3. Which traditional eating behaviors are more pronounced in urban older adults, and which are more pronounced in rural older adults?

Answering these questions will provide detailed information about a broad range of facets of traditional and modern eating in an under-researched sample. This might hint towards signs of modernization in this area of India as well as fuel further research on this topic across different regions in India. Altogether, this might provide some basis for future research on traditional eating, cultural identity, and well-being.

## Methods

### Sample and procedure

In total, 120 participants took part in this study (see section Analytical Procedure for sample size calculation). Sample characteristics and differences between urban and rural participants are displayed in Table 1. The urban (*n* = 60) and rural (*n* = 60) sample did not differ in gender composition. However, the urban sample was slightly older than the rural sample and was comprised of a higher proportion of Hindus. Also, they reported better education, higher socio-economic status, and a greater proportion of higher caste membership, which mirrors a general difference in caste distribution and measures of socio-economic status, such as education or income, between urban and rural areas in India (Agrawal, 2014; Hnatkovska & Lahiri, 2013). The sample, compared with population data, consisted of slightly more females (55% vs. 48% in Gujarat) and slightly fewer Hindus (87% vs. 89% in Gujarat, Directorate of Census Operations, Gujarat, 2011). Moreover, the sample was more educated compared to older adults in Gujarat (19% vs. 59% below primary education; 34% vs. 16% primary education; 24% vs. 20% secondary education; 23% vs. 6% tertiary education; International Institute for Population Sciences, 2020).

**Table 1.** Socio-demographic characteristics of the sample and differences between the urban and rural sample.

	Overall sample (*n* = 120)	Urban sample (*n* = 60)	Rural sample (*n* = 60)	*χ*^2^ or Welch’s *t*	*df*	*p*	*d^a^ or Phi^b^*
Number of women (%)	66 (55%)	30	36	1.21	1	.271	0.10^b^
Number of men (%)	54 (45%)	30	24				
Mean age in years (*SD*; range)	67.93 (6.42; 60-87)	69.43 (7.00; 60-87)	66.43 (5.45; 60-87)	2.62	111.36	.010	0.48*^a^*
Religion (*n*; %)	Hindu: 105 (87%) Muslim: 13 (11%) Jain: 2 (2%)	Hindu: 58 (97%) Jain: 2 (3%)	Hindu: 47 (78%) Muslim: 13 (22%)	16.15	2	<.001	0.37^b^
Caste membership among Hindus and Jains (*n*; %)	Lower caste: 27 (26%) Higher caste: 77 (74%)	Lower caste: 9 (16%) Higher caste: 48 (84%)	Lower caste: 18 (38%) Higher caste: 29 (62%)	6.79	1	.009	0.26^b^
Mean SES (*SD*; range)	4.66 (1.81; 1-9)	5.82 (1.49; 4-9)	3.50 (1.30; 1-5)	9.09	118	<.001	1.66^a^
Education (*n*; %)	Below primary: 23 (19%) Primary: 41 (34%) Secondary: 29 (24%) Tertiary: 27 (23%)	Below primary: 1 (2%) Primary: 13 (22%) Secondary: 19 (32%) Tertiary: 27 (45%)	Below primary: 22 (37%) Primary: 28 (47%) Secondary: 10 (17%) Tertiary: 0 (0%)	55.46	3	<.001	0.67^b^

*Note.* SES, socio-economic status. Caste membership was assessed with an open-ended question and afterwards categorized into lower and higher castes. The classification of scheduled castes and tribes in Vadodara district from 2011 census was referred for verification. SES was measured with a question adapted from the Cantril ladder where people are asked to rate where they stand in the society in their country from 1 (people with least money, education, and worst jobs) to 9 (people with most money, education, and best jobs). Education as classified by the International Standard Classification of Education 2011 (OECD, 2015).

We recruited participants in Gujarat (Western India) in summer 2019, as part of a larger study investigating traditional and modern eating behavior. We collected urban data from Vadodara city and rural data from nearby villages in Vaghodia taluka, on the outskirts of Vadodara city. Villages had electricity, schools, running water, and drainage facilities. A trained research assistant approached people older than 60 years in these rural and urban areas, and briefed them about the research purpose. Once people agreed to participate, the trained research assistant administered the questionnaire in a face-to-face situation. Specifically, she read the questions to participants and noted their responses. Participants received Rs. 50 for their participation, which approximately equated to one third of the average per capita daily income in Gujarat (average annual per capita income was Rs. 56,802 in 2017/2018; International Institute for Population Sciences, 2020). Participants could choose whether to conduct the survey in English or the local language (Gujarati). Administration of the questionnaire took approximately one hour.

The ethics board of The Maharaja Sayajirao University of Baroda (India) approved the study protocol (2019-12-04). The study conforms to the Declaration of Helsinki. Participants gave written informed consent before completing the survey.

### Measures

We assessed how often or to what degree participants performed 57 traditional and modern eating behaviors. We based the selection of the 57 behaviors on results of a previous survey study (Sproesser et al., 2022) to ensure that they are part of traditional and modern eating in the context of India. Specifically, Sproesser et al. (2022) asked a sample of 550 Indians from four regions of India (North, East, South, West) to rate the traditionality and modernity of 86 facets of traditional and modern eating behavior, which were compiled through an extensive literature review and expert discussions (Sproesser et al., 2019). More precisely, participants were asked to rate the degree to which these facets represent traditional and modern eating of the majority of people living in the West of India (around Vadodara, Gujarat) (wording for data collection in Western India) on a 7-point scale from −3 ‘very traditional’ to +3 ‘very modern’. A hierarchical cluster analysis revealed that the four regions showed a highly similar pattern, which were, thus, aggregated. With a value of −1 meaning ‘slightly traditional’, facets were classified as part of traditional eating if they had a mean of −0.5 or lower. Equally, with a value of 1 meaning ‘slightly modern’, facets were classified as part of modern eating if they had a mean of 0.5 or higher. Accordingly, the Indian sample rated 63 facets to be part of traditional and modern eating behavior in India (Sproesser et al., 2022). As the present study was part of a larger project, the number of items to include in the survey was limited. Therefore, facets with low traditionality or modernity were omitted.

Items are displayed in Tables 2 and 3. Participants responded on a 7-point rating scale (−3 = never/strongly disagree, 0 = sometimes/neither nor, 3 = always/strongly agree). The 57 facets and corresponding items cover both the dimensions of *what* and *how* people eat. The ‘what’ dimension includes six subdimensions – Ingredients, Processing, Preparation, Temporal Origin, Spatial Origin, and Variety – and the ‘how’ dimension includes six other subdimensions – Temporal Aspects, Spatial Aspects, Social Aspects, Meals, Appreciation, and Concerns.

**Table 2.** Modern eating items and results of linear regressions with modern eating items as dependent variable and urban-rural as independent variable (*n* = 120).

Item	*B^a^*	*SE*	*t*	*p*	*η^2^_p_*
1. Using plastic utensils while eating (e.g. plastic forks) (A)	−0.22	0.10	−2.24	.027*^b^*	.041
2. Eating foods from other countries’ cuisines (TO)	−0.38	0.09	−4.30	<.001	.135
3. Drinking soft drinks (e.g. cola) during the main meal (M)	−0.38	0.09	−4.34	<.001	.138
4. Eating food from vending machines (e.g. chips) (SO)	−0.58	0.14	−4.32	<.001	.136
5. Eating foods that are imported from all over the world (SO)	−0.47	0.11	−4.42	<.001	.142
6. Eating frozen meals (Proc)	−0.57	0.12	−4.66	<.001	.155
7. Consuming diet drinks or foods (I)	−0.50	0.14	−3.48	.001	.093
8. Eating while working (SA)	0.33	0.12	2.82	.006	.063
9. Buying foods in supermarkets or chain stores (SO)	−0.52	0.12	−4.37	<.001	.139
10. Using time-saving food preparation equipment such as microwave ovens (Prep)	−0.88	0.18	−5.00	<.001	.175
11. Eating take-away or delivered meals (Prep)	−0.25	0.17	−1.47	.143	.018
12. Eating fast food (e.g. hamburgers) (Proc)	−0.38	0.17	−2.21	.029*^b^*	.040
13. Eating alone outside of home (Soc)	−0.40	0.17	−2.35	.020	.045
14. Eating pizza (TO)	−1.00	0.19	−5.40	<.001	.198
15. Consuming artificial sweeteners (e.g. aspartame in diet drinks, to sweeten coffee or tea) (I)	−0.42	0.22	−1.90	.060	.030
16. Eating ready-to-eat foods (e.g. premade sandwiches) (Prep)	−0.58	0.18	−3.23	.002	.081
17. Eating at buffet or all-you-can-eat restaurants (SA)	−0.62	0.13	−4.92	<.001	.170
18. Eating while being conscious of calorie content or nutritional value (C)	−0.77	0.23	−3.35	.001	.087
19. Eating foods that are industrially mass produced (Proc)	−0.57	0.21	−2.70	.008	.058
20. Throwing away food (A)	0.02	0.17	0.10	.924	.000
21. Drinking soft drinks (e.g. cola) (Proc)	0.02	0.20	0.08	.934	.000
22. Eating while walking/ traveling from one place to another (SA)	−0.23	0.15	−1.59	.116	.021
23. Being concerned about eating too much (C)	−0.25	0.20	−1.27	.208	.013
24. Eating out of home (SA)	−0.61	0.17	−3.63	<.001	.101
25. Eating foods that are recently produced; i.e. new foods that were not eaten previously (before 1940) (TO)	0.35	0.21	1.66	.101	.023
26. Eating industrially processed foods (e.g. chips, ready-made meals) (Proc)	−0.17	0.26	−0.63	.528	.003
27. Doing something else while eating (e.g. watching television) (A)	−0.90	0.44	−2.05	.042*^b^*	.035
28. Choosing food according to individual preferences rather than social norms (Soc)	−0.73	0.30	−2.41	.017*^b^*	.047
29. Food is readily available wherever I am during the day (e.g. when going to work). (SO)	−0.50	0.14	−3.71	<.001	.104
30. All my foodstuff is purchased (as opposed to grown or raised by myself). (SO)	0.15	0.25	0.59	.554	.003

Note. Related subdimensions are written in parentheses after items. ^a^Positive values indicate larger means for the rural participants. ^b^Please note that the differences became non-significant when including age or religion in the regressions.

Abbreviations: TO, Temporal Origin; I, Ingredients; Soc, Social Aspects; SA, Spatial Aspects; SO, Spatial Origin; Proc, Processing; C, Concerns; Prep, Preparation; A, Appreciation; M, Meals.

**Table 3.** Traditional eating items and results of linear regressions with traditional eating items as dependent variable and urban-rural as independent variable (*n* = 120).

Item	*B^a^*	*SE*	*t*	*p*	*η^2^_p_*
1. Drinking water (I)	−0.08	0.08	−1.00	.319	.008
2. Eating grains (e.g. wheat, rice, corn) and grain products (e.g. bread) (I)	−0.05	0.14	−0.36	.716	.001
3. Eating vegetables (I)	0.00	0.16	0.00	1.00	.000
4. Eating legumes (e.g. beans, lentils) (I)	0.00	0.17	0.00	1.00	.000
5. Eating meals cooked or prepared at home (Prep)	−0.28	0.08	−3.36	.001	.087
6. In my family, I eat the main meal at home at the same time as the others. (TA)	0.02	0.20	0.08	.934	.000
7. Eating at home (SA)	−0.30	0.10	−2.96	.004	.069
8. At home, women do all the cooking. (Prep)	0.48	0.17	2.86	.005	.065
9. Eating basic foods like corn or rice (I)	−0.48	0.19	−2.62	.010	.055
10. When eating with other people at home: eating the same foods as the others (Soc)	−0.03	0.23	−0.15	.884	.000
11. Eating fruits (I)	−0.57	0.26	−2.17	.032	.038
12. Eating seasonal foods (SO)	−0.62	0.10	−5.99	<.001	.233
13. Eating dishes that are typical for the West of India (around Vadodara, Gujarat) (TO)	−0.40	0.15	−2.65	.009	.056
14. Eating dairy products (e.g. milk, cheese, yoghurt) (I)	−1.28	0.17	−7.49	<.001	.322
15. Eating at fixed mealtimes (TA)	−0.32	0.22	−1.43	.156	.017
16. Eating foods that are produced in the region (SO)	−1.60	0.22	−7.23	<.001	.307
17. I eat a large variety of fruits and vegetables. (V)	−0.72	0.18	−4.04	<.001	.121
18. Larger family events center on meals (Soc)	−0.93	0.23	−4.02	<.001	.121
19. I eat in a way that shows respect for the others. (A)	−1.37	0.25	−5.55	<.001	.207
20. I appreciate food. (A)	−0.50	0.17	−3.02	.003	.072
21. I know how to cook. (Prep)	0.37	0.44	0.84	.402	.006
22. Eating while being served food by others (Soc)	2.02	0.27	7.45	<.001	.320
23. Eating food that has been prepared in grandmother’s way (Prep)	1.22	0.24	5.03	<.001	.176
24. Men get preferential treatment over women at my mealtimes. (Soc)	0.65	0.31	2.13	.035	.037
25. Eating meals that end with a sweet dessert (M)	−0.07	0.21	−0.33	.743	.001
26. Taking time preparing food (Prep)	−0.55	0.19	−2.89	.005	.066
27. Eating eggs (I)	0.92	0.26	3.55	.001^b^	.097

Note. Related subdimensions are written in parentheses after items. ^a^Positive values indicate larger means for the rural participants. ^b^Please note that the difference became non-significant when including religion in the regression.

Abbreviations: TO, Temporal Origin; I, Ingredients; Soc, Social Aspects; SA, Spatial Aspects; TA, Temporal Aspects; SO, Spatial Origin; V, Variability; Prep, Preparation; A, Appreciation; M, Meals.

We measured socio-economic status using a question adapted from the Cantril Ladder (Cantril, 1965), where people are asked to rate where they stand in the society in their country from 1 (people with least money, education, and worst jobs) to 9 (people with most money, education, and best jobs). Participants indicated their highest level of education in line with the International Standard Classification of Education 2011 (OECD, 2015)- i.e. 0 ‘Early childhood education’, 1 ‘Primary education’, 2 ‘Lower secondary education’, 3 ‘Upper secondary education’, 4 ‘Post-secondary non-tertiary education’, 5 ‘Short-cycle tertiary education’, 6 ‘Bachelor’s or equivalent level’, 7 ‘Master’s or equivalent level’, 8 ‘Doctoral or equivalent level’.

We created the survey in English and translated it into Gujarati using the back-translation method (Brislin, 1970). Differences between the original and back-translated version were discussed and resolved by joint agreement of translators.

### Analytical procedure

We conducted statistical analyses using IBM SPSS (Version 27 for Windows) and imputed missing data in traditional and modern eating items using the Expectation Maximization algorithm in SPSS (Gold & Bentler, 2000). Missing values were below 2% for all imputed variables.

To investigate which facets of traditional eating are enacted rarely and which facets of modern eating are enacted often, we tested which traditional eating facets were significantly lower than the middle of the scale (0) and which modern eating facets were significantly higher. We chose the middle of the scale as the cut-off because values above this reflect behaviors that are enacted often or always or participants agreed to enact the behavior. Also, values below this mean that behaviors are enacted rarely or never or participants disagreed to enact the behavior. We conducted one sample t-tests for slightly skewed variables, and conducted sign tests for extremely skewed variables (skewness higher than 2), as suggested by Riehl and Wilkie (1996; see also Vorberg & Blankenberger, 1999).

To test whether rural and urban older adults significantly differed from each other in traditional and modern eating, we conducted the following analyses. As the assumptions of normality and equal variances were often not met, we conducted linear regressions with robust standard errors using the HC4 estimator (Hayes & Cai, 2007). Specifically, we computed a linear regression for each of the traditional and modern eating facets as dependent variable and urban vs. rural as independent variable. Moreover, we conducted control analyses, as the rural and urban sample significantly differed in age and religion. Specifically, we added each of the control variables age and religion as second independent variable to the linear regressions. We dummy-coded categorical variables and *z*-standardized continuous variables before including them in the regressions. For religion, we only took Hindus and Muslims into account because of the small cell size of Jains in our sample. We did not perform control analyses for caste membership, SES, and education because the differences between our rural and urban sample appeared to mirror general differences between urban and rural areas in India (Agrawal, 2014; Hnatkovska & Lahiri, 2013). To further secure the results, we also performed non-parametric analyses, which revealed largely comparable results (see Tables S3 and S4 in the Supplement).

We set a significance level of .05 (two-tailed) for all tests. As the results of all the individual statistical tests were important, we did not apply corrections for multiple testing, as suggested by Armstrong (2014; cf., Nakagawa, 2004). To determine the required sample size, we conducted a power analysis with G*Power 3.1.9.7 (Faul et al., 2009, 2007). The most statistical power was needed to detect differences between the urban and rural sample in the linear regressions. Specifically, setting *α* = .05 and power = .80 revealed a minimum sample size of 115 participants to detect a small to medium effect.

## Results

### Overall level of traditional and modern eating behaviors in older adults living in an area of Gujarat, Western India

**Figure 1.**
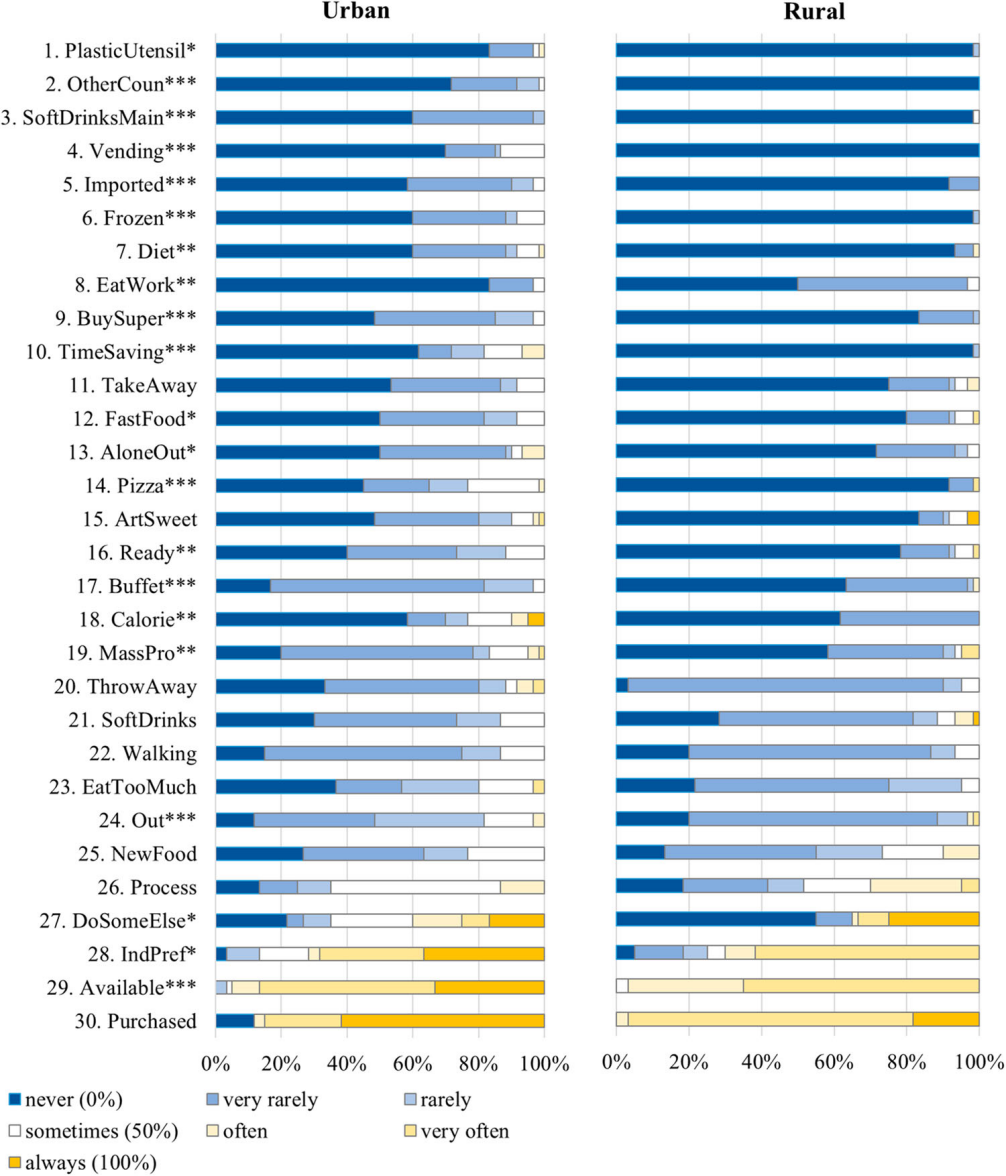
Percentage of participants who responded to show the modern eating behaviors from never to always for the urban and rural samples (*n* = 120). *Not*e. Items are sorted by their overall mean in ascending order. Full item names are displayed in Table 2. * *p* < .05; ** *p*  < .01; *** *p*  < .001.

**Figure 2.**
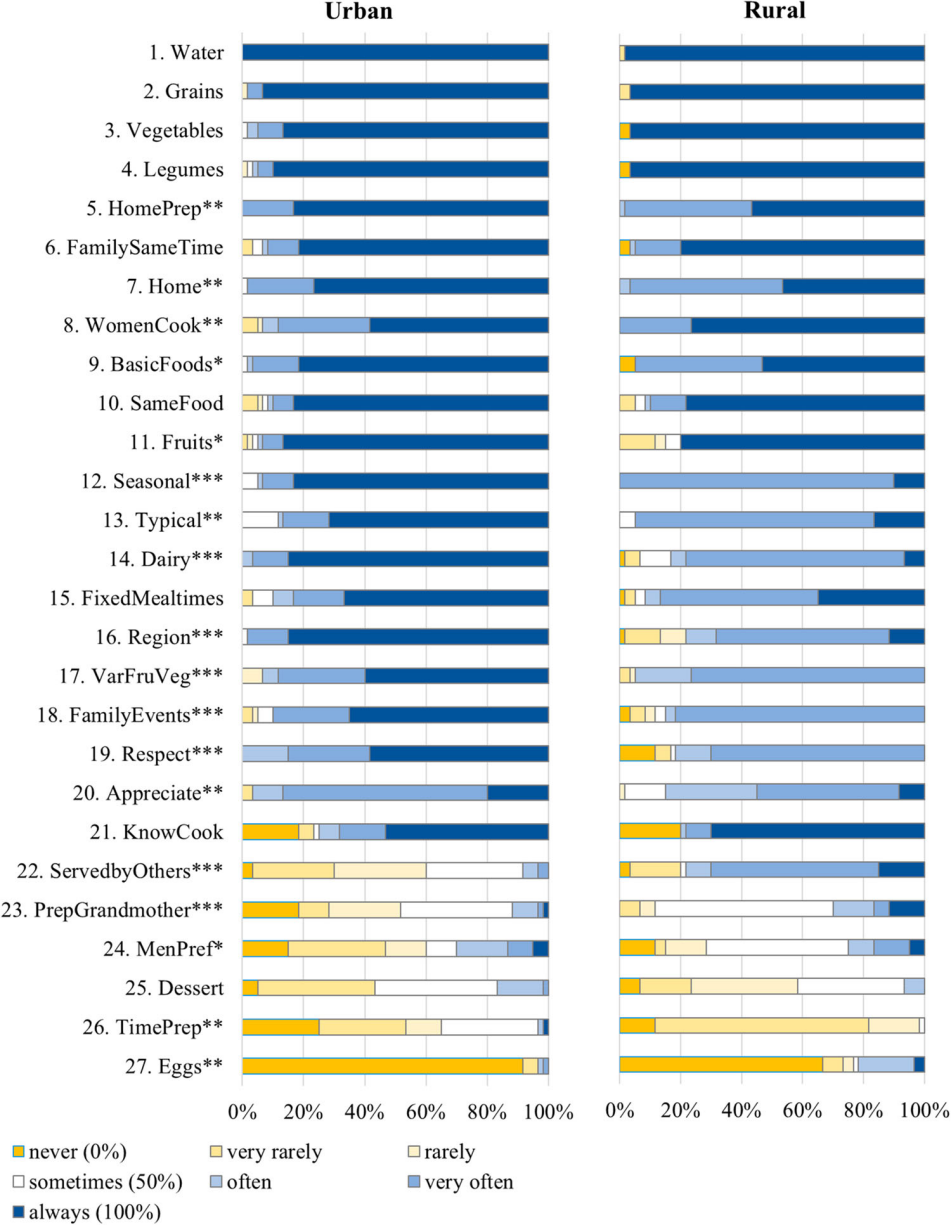
Percentage of participants who responded to show the traditional eating behaviors from never to always for the urban and rural samples (*n* = 120). *Not*e. Items are sorted by their overall mean in descending order. Full item names are displayed in Table 3. * *p* < .05; ** *p*  < .01; *** *p*  < .001.

Descriptive statistics for the traditional and modern eating items are displayed in Tables S1 and S2 in the Supplement. Figures 1 and 2 present the percentage of participants who indicated that they enact the eating behaviors from never to always, for both the urban and rural samples. Figures S1 and S2 in the Supplement present means for the traditional and modern eating items for the urban vs. rural sample, separating men and women.

With regard to traditional eating, means of the following items were significantly lower than the middle of the scale (0) and were thus disagreed to or enacted rarely: ‘Men get preferential treatment over women at my mealtimes’, *M* = −0.41, *SD* = 1.69, *t*(119) = −2.64, *p* = .005, *d* = 0.24; ‘Eating meals that end with a sweet dessert’, *M* = −0.77, *SD* = 1.16, *t*(119) = −7.24, *p* < .001, *d* = 0.66; and ‘Taking time preparing food’, *M* = −1.64, *SD* = 1.08, *t*(119) = −16.72, *p* < .001, *d* = 1.53. For the item ‘Eating eggs’, the proportion of participants with values lower than the middle of the scale (0) was significantly larger than the proportion of participants with values higher than the middle of the scale (87% vs. 13%; *p* < .001). Thus, these four facets were enacted at a low level.

With regard to modern eating, means of the following items were significantly higher than the middle of the scale (0) and were thus agreed to or enacted often: ‘Choosing food according to individual preferences rather than social norms’, *M* = 1.20, *SD* = 1.70, *t*(119) = 7.74, *p* < .001, *d* = 0.71; and ‘Food is readily available wherever I am during the day’, *M* = 1.87, *SD* = 0.78, *t*(119) = 26.31, *p* < .001, *d* = 2.40. For the item ‘All my foodstuff is purchased’, the proportion of participants with values higher than the middle of the scale (0) was significantly larger than the proportion of participants with values lower than the middle of the scale (94% vs. 6%; *p* < .001). Thus, these three behaviors were enacted at a high level. Interestingly, the four traditional and three modern eating facets which showed relatively low and high values represent both what people eat (subdimensions Ingredients, Preparation, and Spatial Origin) as well as how people eat, (subdimensions Meals and Social Aspects; see Tables 2 and 3; Sproesser et al., 2019, 2022, 2018).

### Which traditional and modern eating behaviors differ between the urban and rural sample?

Concerning modern eating, analyses revealed that for 20 out of 30 modern eating items, urban older adults showed significantly higher values than rural older adults (see Table 2 and Figure 1). However, four of these 20 differences became non-significant when including age or religion in the regressions (see Table 2 and Tables S5–S8 in the Supplement). The 16 items that still showed significantly higher values in the urban sample cover a broad range of nine different subdimensions. In contrast, rural older adults showed significantly higher values than urban older adults for one out of 30 items – eating while working. This difference remained significant when including age or religion in the regressions. Hence, for most modern eating facets, urban older adults showed higher values than rural older adults.

Concerning traditional eating, analyses revealed that for five out of 27 traditional eating items, rural older adults showed significantly higher values than urban older adults (see Table 3 and Figure 2). Specifically, rural older adults had significantly higher vales than urban older adults for women doing all the cooking (item no. 8), eating while being served food by others (item no. 22), eating food that has been prepared in grandmother’s way (item no. 23), and men getting differential treatment at mealtimes (item no. 24), covering two different subdimensions. The significantly higher value for eating eggs (item no. 27) in the rural sample disappeared, however, when including religion into the regression (see Table S6 in the Supplement). In contrast, urban older adults showed significantly higher values than rural older adults for 13 out of 27 items, covering eight different subdimensions. These differences remained significant when including age or religion into the regressions. Hence, a rather mixed picture emerged with regard to urban-rural differences in traditional eating facets.

## Discussion

The present study investigated traditional and modern eating behavior in older adults living in an area of Gujarat, Western India. Specifically, we examined (a) which facets of traditional eating are enacted rarely and which facets of modern eating are enacted often; (b) whether modern eating is more prevalent among urban than rural older adults; and (c) which traditional eating behaviors are more pronounced in rural older adults, and which are more pronounced in urban older adults. Overall, our sample of older Gujaratis reported a high level of traditional eating behaviors and a low level of modern eating behaviors. However, they reported a low level of the traditional eating facet of men getting preferential treatment and a high level of the modern eating facet of food being readily available. Moreover, most modern eating facets were more pronounced in the urban than in the rural sample. This was also the case for half of all traditional eating facets. Interestingly, however, the rural sample displayed higher values for four traditional eating facets– mostly for those facets displaying a generally low level. Altogether, results may suggest some signs of modernization among older adults in this area of Gujarat with regard to changing gender roles and better food availability. However, decreases in traditional eating behaviors might be less pronounced in rural than in urban older adults in this area of Gujarat. Furthermore, our sample of older Gujaratis did not seem to be exempt from displaying more modern eating behaviors in urban than in to rural areas. At the same time, however, better food availability might also allow urban older Gujaratis to show more traditional eating behaviors.

### Traditional and modern eating among older adults in an area of Gujarat, Western India

The generally high level of traditional eating behaviors and low level of modern eating behaviors among our sample of older Gujaratis is in line with other work that points towards a traditional and less modern eating in older adults (Imamura et al., 2015; Pingali & Khwaja, 2004; Popkin et al., 2012) and a comparatively high level of food neophobia (Siegrist et al., 2013). Still, seven traditional and modern eating facets showed relatively low and high levels. This might hint towards first signs of change in older adults in this area of India. Interestingly, these facets represent both the dimensions *what* and *how* people eat. More precisely, these facets belong to the five subdimensions Ingredients, Preparation, Meals, Social Aspects, and Spatial Origin (see Tables 2 and 3; Sproesser et al., 2019, 2022). One explanation for this finding might be that changes might occur not only with regard to the largely studied nutrients or food groups (e.g. Law et al., 2020; Misra et al., 2011; Siddiqui et al., 2019), but also with regard to how people eat.

Our cross-sectional design allows us to examine which traditional and modern eating facets show a comparatively low and high level, which might indicate broader changes. Future longitudinal research is needed, however, to uncover any ongoing changes. For instance, the low consumption of eggs and sweet desserts might either be a sign of less traditional eating than in the past, or eggs and sweet desserts might have been consumed rarely already in the past for reasons of religious and caste restrictions, availability, or because not every Indian meal ends traditionally with a sweet dessert (Sen, 2009). In a similar vein, the reported low frequency of taking time for food preparation, even among women (see Figure S2 in the Supplement), might mirror a decrease in time for food preparation due to an increased availability of kitchen gadgets or cooking gas as compared to fire wood (Department for International Development, 2010). Such a decrease in time for food preparation has also been reported, for example, in the USA (Smith et al., 2013). Alternatively, it might result from others, e.g. daughters(-in-law), being mostly responsible for meal preparation (Sen, 2009). Also, the high level of individual preferences driving food selection might either be a sign of an increasing individualization of eating behavior (cf. Fischler, 2011), or perhaps our sample underestimated the impact of social norms, which might act at a more implicit level (cf., Robinson et al., 2011). Still, the finding that our sample tended to disagree that men get preferential treatment might indicate changing gender norms. This would be in line with findings of declining gender inequality between 2000 and 2018 in India (Tisdell, 2021). Moreover, the reported high level of food availability might mirror some progress in food security. Specifically, food availability is a component of food security that has increased slightly within the past 30 years (Reddy, 2016). The high level of purchased foods (as opposed to grown or raised by oneself) is in line with changing food environments and increasing reliance on supermarkets, in both urban and rural areas (Vicol, 2018).

### Differences between urban and rural older adults in an area of Gujarat, Western India

With regard to modern eating, our results extend previous work (e.g. Hawkes et al., 2017; Popkin et al., 2012; Shetty, 2002) by providing some evidence for a rural-urban difference among a sample of older adults living in an area of Gujarat and for a broad range of eating facets. Specifically, for 16 out of 30 modern eating facets, representing nine subdimensions (see Table 2), the urban sample had significantly higher values than the rural sample, such as for eating out of home (see also Smith, 2015). In contrast, eating while working was more pronounced in the rural than in the urban sample. This might be the result of a higher percentage of rural Indians who still work despite older age (e.g. in agriculture), as rural older adults often have no retirement age or pension benefits (Banerjee, 2021). Hence, it would be useful to investigate whether this difference reverses among younger urban and rural Indians.

Interestingly, no significant difference emerged between the urban and rural sample for 13 modern and ten traditional eating facets, such as for the consumption of processed foods (cf., Pandey et al., 2020). There are at least two potential explanations for the absence of these differences. First, for some eating facets, older age might have prevented our sample of urban older adults from showing less traditional and more modern behaviors. This would be in line with the assumption that older adults are generally less affected by the modernization of eating behavior (Pingali & Khwaja, 2004; Popkin et al., 2012). This explanation might be especially the case for modern eating facets that display a relatively low overall level, such as eating take-away or delivered meals, as well as for traditional eating facets with a high overall level. Second, the missing differences might mean that our rural sample has already caught up with modernization trends for those eating facets. This might be especially the case for modern eating facets with a relatively high overall level, such as purchasing all foodstuffs, as well as for traditional eating facets with low overall level. As such, future research with longitudinal designs is needed to examine modern eating trends in both urban and rural older adults.

With regard to traditional eating, urban-rural differences occurred in both directions. In line with the assumption that more modern eating goes along with less traditional eating, we found that the urban sample showed less traditional eating behaviors than the rural sample with regard to four facets. Notably, three of these differences occurred for the six eating facets that showed the lowest overall level. If one assumes that a low overall level indicates some signs of modernization, this might mean that the urban sample showed more modernization for these changing facets. Again, however, our cross-sectional design can only hint at possible changes, and longitudinal work is needed to uncover which changes indeed occurred.

For 13 traditional eating facets, the urban sample displayed higher values than the rural sample. Better food security, specifically higher food availability, and fewer problems with hunger in urban as compared to rural areas (Kumar et al., 2012) might explain this pattern. More precisely, when more food is available in urban than in rural areas, this might explain the higher consumption of traditional foods in the urban sample, such as fruits or dairy products. Moreover, the finding that the urban sample reported eating more often at home, eating more home-prepared foods, and more often taking time for food preparation might again be explained by more rural Indians still working despite older age (Banerjee, 2021). Lastly, some of the differences might also be due to better understanding of abstract terms such as showing respect, appreciation, or eating typical dishes in the urban, more educated sample than in the rural, less educated sample (cf., Dean et al., 2005).

### Limitations

There are several limitations to this study. First, the assessment of traditional and modern eating was based on a previous study, asking a sample of 550 Indians about the traditionality and modernity of a set of facets (Sproesser et al., 2022). However, this basis represents only one piece of evidence and more research is needed to more fully understand what constitutes traditional and modern eating in India. Second, our sample was of rather small size, higher than average educated, and recruited from only one area of Gujarat. Hence, results might not be generalizable to people with lower education, or from other regions. Third, rural participants were recruited in villages relatively close to Vadodara city. Hence, it remains open whether our results replicate for older adults living in more remote rural areas. Fourth, our study solely relied on self-report measures, which might be prone to differential understanding (cf., Dean et al., 2005) and memory biases (Renner et al., 2015). In addition, responses in face-to-face situations might be biased through social desirability (Gupta & Thornton, 2002). However, more ‘anonymous’ methods such as online surveys would not have been feasible for this sample, especially to reach rural older Indians with comparatively low SES (Aubert & Friedrich, 2021). Still, future research using, for example, behavioral observation is needed to complement the results of the present study.

### Conclusion and implications

The present paper is a first step towards better understanding the extent of traditional and modern eating behavior of the under-researched but growing subpopulation of older Indian adults (Schrock et al., 2017; Shetty, 2002). The traditional and modern eating facets that were reported at a low and high level in this sample represent both *what* and *how* people eat, and span several subdimensions. Thus, this might indicate that eating behavior in Gujarat might be changing on a broader level than just with regard to nutrients and food groups. This underlines the need for future longitudinal studies to include a broader assessment of the multiple dimensions of traditional and modern eating behavior that goes beyond nutrients and food groups.

With regard to cultural identity, the overall high level of traditional and low level of modern eating in our sample might be beneficial for well-being (cf., Guerrero et al., 2010; Tartakovsky & Abu Kheit, 2017; Usborne & Taylor, 2010). At the same time, leaving behind some characteristics of traditional eating, such as men getting preferential treatment, and an increase in some characteristics of modern eating, such as high food availability, might be further beneficial. Specifically, ‘gender equality’ and ‘zero hunger’ are two of the Sustainable Development Goals by the UN (UN, 2022). Moreover, our finding that the rural sample tended to show less traditional eating, might mirror less food security than in the urban sample (Kumar et al., 2012). As food insecurity has been related to stress, depression and even suicidal ideation in older adults from low- and middle-income countries (Selvamani & Arokiasamy, 2021; Smith et al., 2021, 2022), it seems desirable to further promote high food availability, especially in rural areas of India.

## Data Availability

Data are available upon request from the lead author.

## Geolocation information

Data were collected in Gujarat, India.
